# A Case of Using No-Touch Saphenous Vein Graft in Redo CABG after Multiple Failed Percutaneous Coronary Interventions

**DOI:** 10.21470/1678-9741-2021-0203

**Published:** 2022

**Authors:** Victor Edin, Håkan Geijer, Piotr Jakuszewski, Domingos Souza

**Affiliations:** 1 Department of Vascular and Cardiothoracic Surgery, Faculty of Medicine and Health, Örebro University, Örebro, Sweden.; 2 Department of Radiology, Faculty of Medicine and Health, Örebro University, Örebro, Sweden.; 3 Department of Nephrology, Faculty of Medicine and Health, Örebro University, Örebro, Sweden.

**Keywords:** Saphenous Vein, Coronary Artery Bypass, Cardiovascular Diseases, Percutaneous Coronary Intervention

## Abstract

The modality of repeat revascularization due to late graft failure is a debated topic. The latest available European guidelines recommend redo coronary artery bypass graft (CABG) for cases of extensively diseased and/or occluded grafts and those with diffuse native vessel disease. We present the case of a patient being relieved of recurrent unstable angina pectoris with redo CABG using no-touch saphenous vein grafts after repeated and unsuccessful attempts with percutaneous coronary intervention (PCI). This could be an alternative to PCI in patients with a complex medical history. Teamwork between cardiologists and surgeons is pivotal in deciding the best treatment modality.

**Figure:**
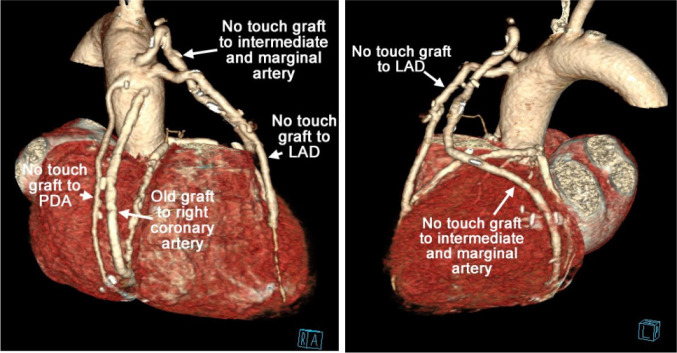

**Table:** 

Abbreviations, acronyms & symbols	
CABG	= Coronary artery bypass graft
CT	= Computed tomography
LAD	= Left anterior descending artery
PCI	= Percutaneous coronary intervention
PDA	= Posterior descending artery
RCA	= Right coronary artery
SVG	= Saphenous vein graft

## INTRODUCTION

Repeat revascularization due to late graft failure is a debated topic. Limited data from previous studies leave clinicians to decide between redo coronary artery bypass graft (CABG) and percutaneous coronary intervention (PCI). The latest available guidelines from the European Society of Cardiology and the European Association of Cardiothoracic Surgeons recommend redo CABG for cases of extensively diseased and/or occluded grafts and those with diffuse native vessel disease^[1]^. We present the case of a patient being relieved of recurrent unstable angina pectoris following redo CABG using no-touch saphenous vein grafts after repeated and unsuccessful attempts with PCI.

## PROCEDURE

This case is about a 71-year old male with a previous history of type 1 diabetes mellitus complicated with blindness. He also suffered from chronic renal failure and peripheral arterial disease, resulting in toe amputations.

**Fig. 1:**
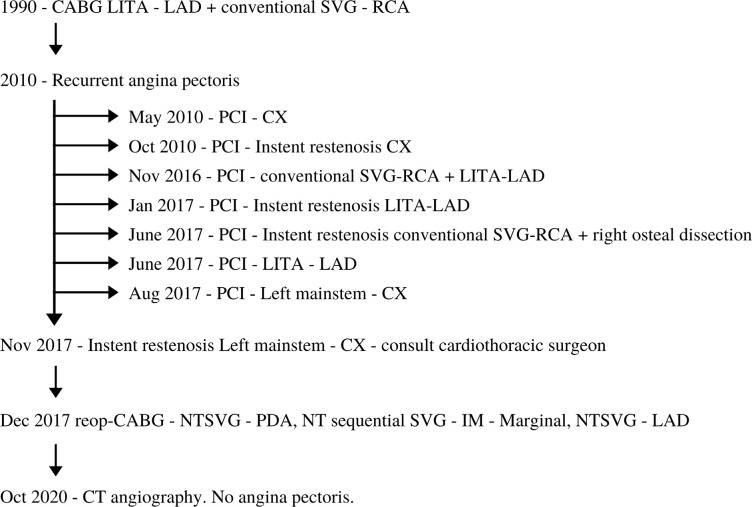
Flowchart of the revascularization treatment of the patient. CABG=coronary artery bypass graft; Cx=circumflex artery; LAD=left anterior descending artery; LITA=left internal thoracic artery; NT=no-touch; NTSVG=no-touch saphenous vein graft; PCI=percutaneous coronary intervention; RCA=right coronary artery

**Fig. 2:**
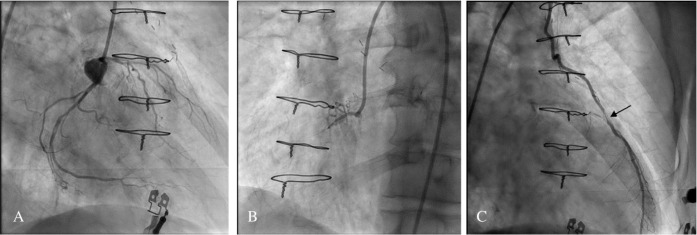
(A) Preoperative coronary angiography, right coronary. (B) Preoperative coronary angiography, left coronary. (C) Preoperative coronary angiography, LITA to LAD. Note the tight stenosis at the anastomotic site (arrow).

In 1990, at age 41, he underwent CABG surgery due to angina pectoris and received a saphenous vein graft (SVG) to the right coronary artery (RCA) and the left internal thoracic artery to the left anterior descending artery. Until 2010 he was relieved of angina pectoris. Between 2010 and 2017, due to recurrent angina pectoris, seven PCI procedures to both the SVG, left internal thoracic artery and native coronary arteries were made, four of which during 2017 (Figure 1). In November 2017, at age 68, he was readmitted due to a new episode of unstable angina pectoris and the angiographic assessment showed in-stent restenosis in all previously treated vessels (Figure 2 and preoperative coronary angiography in supplementary material (Video 1). Due to repeated unsuccessful PCI treatments within a short period of time leading to recurrent episodes of unstable angina, contact was established with the Department of Cardiothoracic Surgery. The decision was made to perform redo CABG surgery, but now using SVG harvested with the no-touch technique. During this procedure, the SVG is harvested with a surrounding pedicle to preserve adjacent supporting tissues^[2]^. The patient received a no-touch SVG to the left anterior descending artery (graft flow of 60 ml/min) and a sequential no-touch SVG to an intermediate artery and a marginal artery (graft flow of 65 ml/min), as well as a no-touch SVG implanted to the posterior descending artery (PDA) (graft flow of 27 ml/min).

**Video 1:**
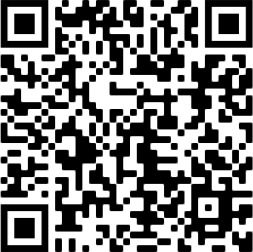
Preoperative coronary angiography showing LITA prior to redo CABG.

Postoperatively, there was a need for extended wound care because of delayed leg wound healing related to the patient’s comorbidities. Diuretics and antibiotics were used. In later postoperative controls, the wound was completely healed and the patient had no complaints.

After a three-year follow-up, the patient was completely asymptomatic. He kept active by using a cycling machine daily, equivalent to 6,000 to 10,000 steps, without any angina pectoris. He described a greatly improved quality of life and was very happy with the surgical outcome. After approval from the ethics committee (2020-02131) and informed consent was obtained from the patient, a follow-up CT coronary angiography was performed, showing that all vein grafts were patent, which corroborated the relief of angina pectoris and well-being of the patient (central picture and three-year follow-up CT coronary angiography (Video 2) in supplementary material).

**Video 2:**
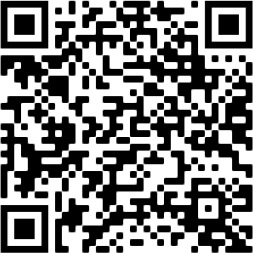
Three-year follow-up CT coronary angiography.

## DISCUSSION

Current guidelines suggest redo CABG in cases of extensive and/or occluded grafts and in those with diffuse native vessel disease, based on studies from the AWESOME randomized controlled studies and registry^[1]^. The use of the internal thoracic artery or other arterial grafts are recommended, if applicable, during the redo procedure. In this case, a patient suffering from multiple comorbidities and use of the left internal thoracic artery during the primary CABG surgery, we did not consider the use of other arterial grafts. The LAD was diffusely diseased and subject to several PCI-stents, which altogether required a longer graft to reach the LAD distally, thus, the use of the right internal thoracic artery was precluded. Furthermore, the risk of mediastinitis in using the right internal thoracic artery was regarded as a greater risk than possible wound issues from the lower leg. Radial grafts were not an option both due to above-described coronary anatomy and also possible future need for a hemodialysis access. Thus, the no-touch saphenous vein graft was considered the best option^[1,3,4]^.

We described a case of successful redo CABG using no-touch SVGs after recurrent unstable angina pectoris repeatedly and unsuccessfully treated with PCI. This case illustrates that reoperation with good quality conduits can be the solution for complicated cases and it is in agreement with current recommendations.

Clearly such cases require close communication and cooperation between cardiologists and surgeons to avoid delays and unnecessary patient suffering in deciding the best treatment modality. In this case, it is even more important since the patient was suffering from chronic renal failure. Hence, excessive use of contrast agents should be avoided. This patient received a total of 470 ml of contrast agent during PCI treatments in 2017, as well as excessive doses of radiation during angiographic assessments in the same year.

**Table:** 

Authors' roles & responsibilities	
VE	Substantial contributions to the conception or design of the work; or the acquisition, analysis or interpretation of data for the work; drafting the work or revising it critically for important intellectual content; final approval of the version to be published
HG	Substantial contributions to the conception or design of the work; or the acquisition, analysis or interpretation of data for the work; drafting the work or revising it critically for important intellectual content; final approval of the version to be published
PJ	Substantial contributions to the conception or design of the work; or the acquisition, analysis or interpretation of data for the work; drafting the work or revising it critically for important intellectual content; final approval of the version to be published
DS	Substantial contributions to the conception or design of the work; or the acquisition, analysis or interpretation of data for the work; drafting the work or revising it critically for important intellectual content; final approval of the version to be published
